# Making the leap from technique to treatment — genetic engineering is paving the way for more efficient phage therapy

**DOI:** 10.1042/BST20231289

**Published:** 2024-05-08

**Authors:** Jessica M. Lewis, Joshua Williams, Antonia P. Sagona

**Affiliations:** School of Life Sciences, University of Warwick, Coventry, U.K.

**Keywords:** clinical trials, host range expansion, phage engineering, phage therapy, phage weaponisation

## Abstract

Bacteriophages (phages) are viruses specific to bacteria that target them with great efficiency and specificity. Phages were first studied for their antibacterial potential in the early twentieth century; however, their use was largely eclipsed by the popularity of antibiotics. Given the surge of antimicrobial-resistant strains worldwide, there has been a renaissance in harnessing phages as therapeutics once more. One of the key advantages of phages is their amenability to modification, allowing the generation of numerous derivatives optimised for specific functions depending on the modification. These enhanced derivatives could display higher infectivity, expanded host range or greater affinity to human tissues, where some bacterial species exert their pathogenesis. Despite this, there has been a noticeable discrepancy between the generation of derivatives *in vitro* and their clinical application *in vivo*. In most instances, phage therapy is only used on a compassionate-use basis, where all other treatment options have been exhausted. A lack of clinical trials and numerous regulatory hurdles hamper the progress of phage therapy and in turn, the engineered variants, in becoming widely used in the clinic. In this review, we outline the various types of modifications enacted upon phages and how these modifications contribute to their enhanced bactericidal function compared with wild-type phages. We also discuss the nascent progress of genetically modified phages in clinical trials along with the current issues these are confronted with, to validate it as a therapy in the clinic.

## Introduction

In the early 1900s, bacterial infections were considered difficult to treat and a likely death sentence. The discovery of penicillin and subsequent antibiotics changed this dramatically, leading to improved health outcomes and life expectancies [1,2]. However, the abuse and overuse of antibiotics coupled with the slow development of novel antimicrobials has led to a new dawn where multidrug-resistant bacteria are prevalent [3]. As we once again face the possibility of bacterial infections being untreatable, the world has been looking for a new hope to overcome the antibiotic crisis. From the ashes of many failed alternatives has emerged an old therapeutic — bacteriophages (phages) [4]. These bacteria-specific viruses were first used as an antibacterial therapeutic over 100 years ago before the mainstream use of antibiotics, but took a backseat when antibiotics became prevalent in the western world. However, their proven history as a therapeutic has led to renewed interest in their usage [5].

Phages are ubiquitous across nature, abundant and diverse and have specificity for their bacterial host, presenting host range that can be narrow or broad, depending on the phage [6]. This review will discuss aspects of lytic and temperate phages [7]. Lytic phages infect host bacteria, replicate rapidly within the cell and eventually induce cell lysis causing the release of the newly produced phage particles [5,6]. Temperate phages similarly infect their host, but rather than replicating and then releasing, they incorporate themselves into the bacterial chromosome as prophages [8]. When the right conditions are met, the temperate phages can be triggered to enter the lytic lifecycle, resulting in release from the host [8]. Due to their ability to lyse the bacteria, lytic phages have been the focus of most of the phage therapy.

While phage therapy has been used successfully to treat multiple bacterial infections, there are limitations that have prohibited widespread use, both from perspectives of safety and from a cost/benefit standard. Despite phages being a natural component of the microbiome, comprising >97% of the gut virome [9], the safety of phage therapy has been a concern. The presence of phage-encoded exotoxins, such as the cholera toxin carried by CTXɸ and the shiga toxins carried by Stx phage, pose a risk to health, as infection of pathogens with these toxin-carrying phages leads to more severe infections [10]. Phages also are known to elicit a response from the human immune system, but this has not been known to cause any adverse effects [11,12]. Another significant concern with lytic phage therapy is due to the lysis of bacteria and consequential mass release of bacterial endotoxins, which are potent activators of the immune system [13,14], but this is unlikely to be problematic as work has shown that the amount of endotoxin released by β-lactam antibiotics is greater than the amount released by lytic phages [15]. Additionally, a comprehensive systematic review has recently shown the safety of these therapeutics, identifying minimal side effects following phage administration [16]. Thus, phage therapy is a promising resource for treating difficult infections.

The enhancement of phages through genetic engineering presents an opportunity for the development of improved viruses with more diverse applications. While the United States Department of Agriculture describes genetic engineering as the ‘Manipulation of an organism's genes by introducing, eliminating or rearranging specific genes using the methods of modern molecular biology, particularly those techniques referred to as recombinant DNA techniques' [17], for the purpose of this review, we will expand the definition to cover techniques that modify the genome, so that it deviates from the wildtype and encompass both targeted modifications and purposeful genetic drift. This review will cover the applications of genetic engineering of phages. For details on how these techniques are performed, readers are referred to the recent review by Mahler et al. [18].

## Increasing host range

**Figure 1.**
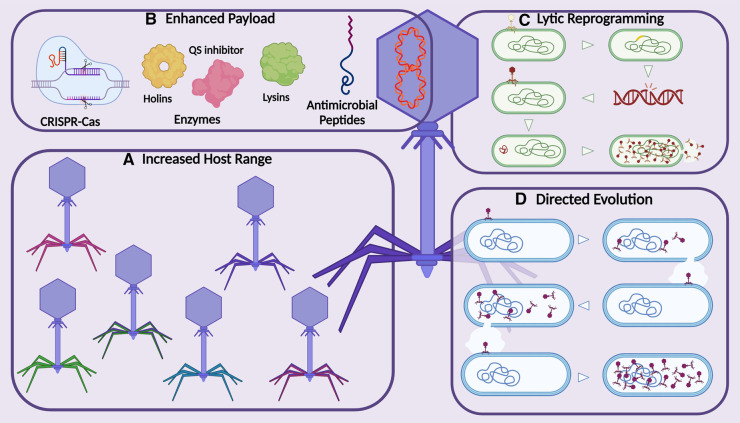
Bacteriophage engineering leads to an improved therapeutic. (**A**) Modification of phage tail fibres through gene replacement or point mutations leads to phages with increased host range. (**B**) The addition of genes to the phage DNA results in the delivery of an enhanced payload, increasing the ability of the phages to target important components within and outside of the bacterial cells, including targeting the cell wall with holins, lysins and antimicrobial peptides, disruption of quorum sensing (QS) with inhibitors and targeting of bacterial DNA with targeted CRISPR–Cas systems. (**C**) The modification of prophages through the inactivation of lysogeny-associated genes results in lytic phages that are suitable for phage therapy. (**D**) Repeated infections of phages against an unexposed host allow for directed evolution to improve the host range and virulence of phages. Created with BioRender.com.

The early therapeutic phage application was designed directly against a particular pathogen [19], ensuring the killing of the causative agent and this was necessary due to the narrow phage host range. Phage host range is typically smaller than the range of narrow-spectrum antibiotics [20], limited to a few isolates and rarely across species for Gram-negatives, whilst narrow-spectrum antibiotics are genus or species specific [21]. For Gram-positives, the phage host range can be slightly broader, with some *Staphylococcal* phages infecting a wide variety of hosts within the same genus [22,23]. The relatively narrow host range means that most phage therapies need to be personalised, as without this, most phages tend to show very little efficacy at clinical trials [24–27]. At a base level, the solution to overcome the narrow host range of phages is to generate a phage cocktail containing multiple phages that target the same bacterial strain [28,29] and phage biobanks have been proposed to aid in the rapid assembly of phage cocktails [30,31]. Genetic engineering has been successfully employed to enhance the host range of phages across many double-stranded DNA families (Figure 1A). Most research has focused on the *Caudoviricetes* class, particularly the *Autographiviridae* and *Straboviridae* families as their highly structured tail that confers host specificity [32] allowed for greater ease of tail modification [33–36]. In particular, the vast majority of studies have focused on a few phages within *Autographiviridae*. The engineering strategies utilise either the complete modification of the tail fibre regions [37–39], the generation of tail fibre chimaeras [33,40,41], exchanges along the tail structure (*Demerecviridae*) [38] or point modifications [35,36]. Point mutation modifications appear to have the most success in randomly expanding the host range through targeting of key tail fibre residues across the phage–bacteria interaction regions. This technique has even allowed for the artificial expansion of phage host range across species, with the tail fibres of the *Escherichia coli* T7 phage being modified to enable also *Yersinia pestis* infection [42]. Despite these successes, the expanded host range *E. coli* phages have not yet made it to the clinic. It is worth noting that there has been instability reported in a T7 *E. coli* phage with a completely switched tail fibre to K1F [39] that changed targeting from a K12 capsule to a K1 capsule strain, resulting in the progeny phage not surviving beyond a few rounds of replication. It is unclear whether this is a result of the tail regions exchanged or due to other replication compatibility factors within the new host strain.

### Enhanced payloads

In addition to broadening the host range, researchers have worked to enhance the payload delivered by phages (Figure 1B). When a phage infects a bacterium, it will deliver its nucleic acid into the cell, hijacking the replication machinery for its own replication. For double-stranded DNA phages, researchers have been able to improve the outcome of phage infections through the addition of genes to encode diverse arrays of cytotoxic payloads [43]. Host DNA is often the target of the modified payload, as degradation of the bacterial DNA restricts the anti-phage defences of the cell [43]. Seminal work by the Marraffini and Lu labs independently published the first enhanced phages in 2014 [44,45]. The initial enhanced phages were designed to carry and deliver a CRISPR–Cas system and guide RNA on a phagemid (a plasmid that can be carried by phage) [44,45] to target important bacterial genes. CRISPR–Cas is a bacterial anti-phage defence system that has subsequently been incorporated into the genome of lytic and temperate phages [44,46–50]. Although the incorporation of different CRISPR–Cas systems has shown increased bacterial killing, with both class 1 and class 2 systems being successfully used, there has been a report of increased phage resistance when the system was used within the temperate *Staphylococcal* phage pKS3 [46]. However, this issue was not observed when the temperate *E. coli* λ phage [48,49] or when a CRISPR–Cas armed *Clostridioides difficile* phage (ɸCD24-2 Δ*lys*) were used [49,50]. Although some resistance was observed against the ɸCD24-2 Δ*lys* phage, albeit less than against the wild-type phage [50], no resistance was noted against the temperate λ phage [49]. Further in support of this strategy, both *E. coli* and *C. difficile* armed phages were successful at treating an infection in a murine model [49,50]. CRISPR–Cas has also been utilised in other animal models; for instance, phage M13 modified to express a CRISPR–Cas13a array targeting carbapenem resistance genes was able to significantly prolong lifespan in a *Galleria mellonella* model when challenged with carbapenem-resistant *E. coli* [51]. The CRISPR–Cas system is quite large and may not be suitable for small phages, but there are minor modifications available, including equipping the phage with antimicrobial peptides [43]. The unsuitability of smaller phages for additional cargo, stems from genome packaging limits. Typically, 102–110% of the phage genome may be packaged by the phage, so the incorporation of genes into larger phages (45 kb) is tolerated, whereas only minimal systems (excluding the CRISPR system) could be added into smaller phages [51,52]. On that note, researchers have begun investigating genome reduction in phages to enhance infectivity. The general principle involves the removal of non-essential genes, often those of unknown function, to generate a derivative with a reduced genome size [53]. Studies generating such phages noted that the antibacterial activity *in vitro* and *in vivo* typically remains unchanged, though some of the deletions had detrimental effects on fitness [53,54]. Genome reduction, however, carries the added advantage of allowing for the insertion of additional genes back into the genome, circumventing packaging restraints. The design of phages that escape antiviral defence mechanisms is also an attractive option to ensure successful lysis and effective treatment. Many bacterial pathogens are armed with defence mechanisms, such as restriction endonucleases and CRISPR–Cas systems, constituting a barrier to phage infectivity [55–57]. Phages have evolved counter mechanisms for these defences, such as protein products that evade or redirect the aforementioned restriction enzymes and CRISPR–Cas systems [58,59]. Such modifications are highly dependent on the defence systems present, requiring rational design of the phage genome tailored to the precise antiviral mechanisms found within the pathogen of interest. Many phages naturally encode antimicrobial peptides, which are known to be beneficial during infection, by enhancing bacterial cell lysis [60–62]; therefore, the addition of these peptides into non-native phages is unlikely to cause disruptions to the surrounding microbiome. A compromise between the small antimicrobial peptides and large CRISPR–Cas genes is the addition of enzyme sequences targeting important components, such as quorum sensing (QS) [63] and the bacterial cell wall [64–66]. Many of these additions aim to replicate enzymatic potential that is naturally delivered by phages [67,68]. Biofilms are one of the bacteria's greatest weapons, building a protective environment that antibiotics and phages alike struggle to penetrate [69]. These extracellular matrices rely on the bacteria's ability to use quorum sensing and to communicate and are composed of polysaccharides, nucleic acid, lipids and proteins, many of which can be targeted by phage-encoded enzymes [70], thus engineered phages may be great weapons to target these. Research has also focussed on the expression and purification of enzymes such as holins, lysins and QS inhibitors [71] to be administered topically [61,72], rather than being engineered into phages. For a comprehensive review of enhanced phage, readers are directed to the review by Schmitt and colleagues [43].

### Lytic reprogramming

In addition to the above, researchers have had success converting temperate phages into lytic via the alteration of genes that facilitate lysogeny [8] (Figure 1C). This conversion can either be done for newly identified temperate phages from environmental sources or from existing prophages identified through online platforms [73,74] within sequenced bacterial genomes. As both lytic and temperate phage are capable of harbouring bacterial host genes within their own chromosome, an important component of phage screenings has been examining phage sequences to avoid using phages encoding toxins or antimicrobial resistance (*amr*) genes through either manual scrutiny or by running phage annotation pipelines that contain built-in *amr* and toxin checks [75–77]. Avoidance of *amr* or toxin carry phages is imperative to avoid enhancing the bacterial target and worsening the infection. As such, removing the integrase or repressor genes from known prophages [73,74] creates derived lytic phages prevented from integrating and forming a lysogen that can be used for the treatment of infections. Despite these technologies, we are not yet seeing this conversion of prophages commonly occurring. However, there have been several notable cases of temperate phages that have been made lytic, particularly the armed ɸCD24-2 Δ*lys C. difficile* phage [50] and the *Mycobacterial* phages that were modified for the treatment of *Mycobacterium abscessus* infections [78,79]. In the latter case, two phages were modified from temperate to lytic through deletion of the *cro* repressor genes and used in a phage cocktail for treatment [78]. The use of these genetically modified phages in human treatment represented the first example of phage therapeutics with gene deletion and these (ZoeJΔ45 and BPsΔ33HTH_HRM10) have been used for further treatment of other *M. abscessus* infections, including in a patient with Cystic Fibrosis [79]. Recently ZoeJΔ45 and BPsΔ33HTH_HRM10 were included in a larger treatment series of 20 patients, with none of the patients experiencing adverse phage-derived effects [80]. These results show the promise of lytic reprogramming as a therapeutic option.

## Directed evolution

Co-evolution of phages and their bacterial hosts has been observed within every ecosystem [81,82], allowing the phages to continuously adapt to the changes of the bacteria and environment. The idea of co-evolution has also been employed to better adapt phages for therapeutic administration, known as ‘Appelmans protocol’ [83] or ‘phage training' [84]. The predominant outcome of the early directed evolution experiments was to produce phage progeny that targets previously insensitive hosts, shifting the lytic ability of the phages to be adapted for new hosts. This is useful for strains where their native phages are difficult to isolate and characterise. This principal has been successfully used to evolve lytic phages (either in cocktails or alone) to obtain optimised activity against *Staphylococcus aureus* (Sb-1) [85], *Pseudomonas aeruginosa* (Pa2, ɸKZ, RWG) [83], *Pseudomonas syringae* (ɸ6, FRS) [86,87], *Shigella flexneri* (Sf6) [88], *E. coli* (P88, pro147, T7) [35,89] and *Klebsiella pneumoniae* (M1) [90] (Figure 1D). Adapted phages (P115, P711, P577) were also raised against Carbapenem-Resistant *Acinetobacter baumannii* (CRAB) using the Appelmans method [91] and while the phages were able to lyse CRAB, there were stability issues that had not been reported in previous studies. While directed evolution is not the fastest modification method, it has the advantage of not requiring previous knowledge of the genetics of the phage and thus can be conducted on newly isolated phages when needed. As phage susceptibility patterns may differ between strains, directed evolution also has the further advantage of producing phage derivatives that are active against strains which were otherwise insensitive to said phage.

## Phage therapy for intracellular pathogens

**Figure 2.**
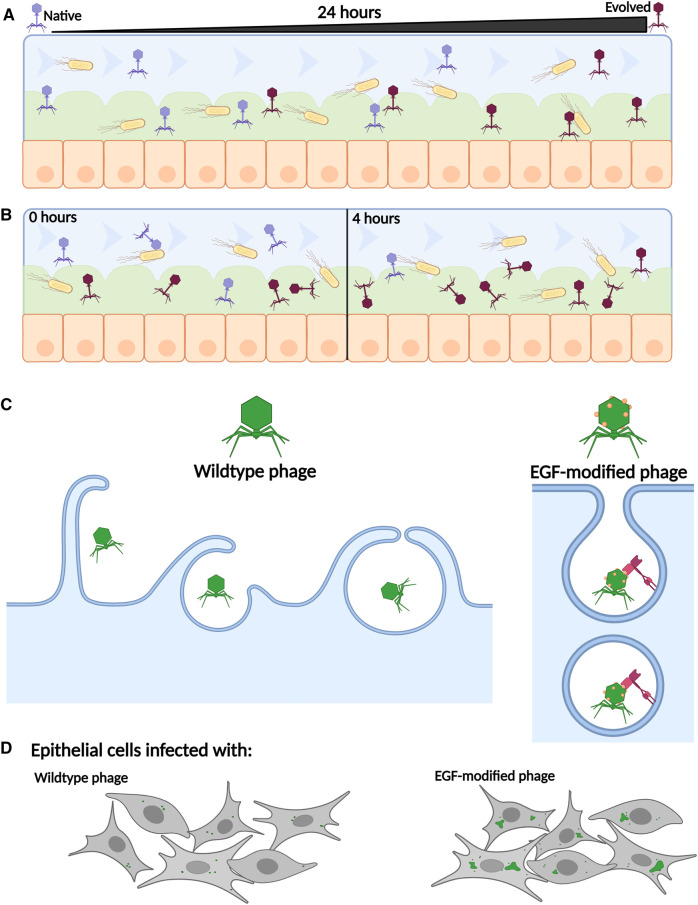
Phages can be modified to enhance their ability to survive and to clear bacteria within a human cell environment. (**A**) When native phages are mixed with bacteria into a dynamic cell environment (Gut-on-a-Chip) for 24 h, they evolve to become more adapt to survival in the mucus (green). (**B**) When equal amounts of native and evolved phages are mixed with bacteria to the dynamic cell environment and left for 4 h, the evolved phages persist better than the native. (**C**) Wild-type phage enters human epithelial cells through micropinocytosis. EGF-modified phage (EGF shown as orange spheres) can bind to the EGF receptor (EGFR — shown in maroon) and will enter cells via endocytosis. (**D**) The phage (green spots) is taken up into endothelial cells at a much greater efficiency when modified (right) compared with wildtype (left). Created with BioRender.com.

One of the most unique applications of phage engineering has been the design of a phage able to interact with human cells alongside its host [92] (Figure 2A). While phages do not directly target human cells, they are capable of entering both phagocytic and non-phagocytic cells in a passive manner, as well as existing within the extracellular environment together with their bacterial host [93–95]. The co-evolution of a T4 phage and *E. coli* in a gut-like environment revealed that the phage was adapting to survive within the mucus layer to increase its adherent ability [92] (Figure 2B). While this study is the first of its kind, the principle should be applied to other environments in the future, as the understanding of the phage adaptions gained in this study will be useful for the engineering of phages for the treatment of pathogens in particular niches. Indeed, work has already begun to modify phages for better survival [96] within their host or to facilitate entry into mammalian cells [97–101]. Similarly to Chin et al., Favor et al. [96] used evolution to increase the thermal stability of *E. coli* and *Salmonella* targeting phages (T3, T7, NBSal001, NBSal002), with the starting phage population having undergone random mutagenesis to enhance the variety of the phages. While the phages in this study were not used in any treatment models, the authors comment that the favoured mutations could be recreated in engineered phages for treatment. For enhanced entry, mammalian cell surface receptors (EFG or FGF2) were incorporated into the capsid of phages [97,99–101] (Figure 2C) or covalently bound to the phage surface [98], which while a simplified process requiring no genetic modification, enabled entry of the first generation of phages. In both circumstances, the receptors increased the uptake into human cell lines [97,98] (Figure 2D) and reduced the bacterial load once inside the human cell [97]. Another strategy that was used to increase the entry into human cells was the use of cell-wall penetrating peptides derived from HIV that were engineered into the capsid of the *Salmonella* phage selz_HA-TAT_ [102]. Similarly to the EFG-modified phage, the penetrating peptide-engineered phage had increased entry into multiple cell types and helped to decrease the burden of bacteria within those cells more than the wildtype [102]. However, these studies have not shown clearance of an infection *in vivo* and have not graduated to trial in a mammalian model. Work in animals is a vital step towards clinical trials and treatment in humans. Moreover, many intracellular pathogens live inside vacuoles within the mammalian cells [103] and the little that is known of the phage lifestyle within cells, assisted or not, suggests that some phages end up in vacuoles targeted for degradation and some in the cytoplasm [94,95]. This trafficking may make it difficult for the phages to reach their intended host within the cells, restricting their efficiency at treating an infection.

### The first engineered phages in clinical trial

The most recent use of genetically engineered phages in treatment is that of the SNIPR0001 clinical trial [41]. SNIPR0001 is the product name for a phage cocktail containing four *E. coli* targeting phages, with activity across a range of clinically isolated *E. coli* strains. These four phages were freshly isolated and belong to the *Tevenvirinae* subfamily and are related to the highly studied T4 phage, allowing for the translation of knowledge and techniques. From this initial mix, one of the phages was modified to obtain an additional tail fibre receptor, resulting in a phage which binds to two cellular targets and thus had extended host range. To further optimise the phage cocktail, the genomes of all four phages were modified to include a CRISPR–Cas array within their genome. This array included a Cas system, as well as spacers to target essential or virulence genes within the *E. coli* chromosome, which allowed for lowered survival rates of the tested *E. coli* strains. The SNIPR0001 study is of importance for phage researchers, as it was the first time that CRISPR-technology had been introduced into the phage genome, with previous studies preferring to use anti-CRISPR systems [104–106]. This is the first documented usage of a weaponised phage, the first engineered phages to enter a clinical trial and only the third documented usage of genetically modified phages as a human therapeutic. The SNIPR0001 phase I clinical trial (NCT05277350) was completed in May 2023, but there has not yet been any notification of the outcome. There has also been discussion of additional CRISPR–Cas3 loaded phages being developed for a clinical trial [107], but as yet this does not seem to have occurred.

## Limitations and conclusions

Despite the advancements made for the engineering and enhancement of phages, there are still many limitations that must be overcome and opportunities that have been missed. Given that antimicrobial resistance is the cause of the renewed interest in phages, there is a lack of research into engineering phages to target main AMR components, such as efflux pumps, which can be shared between strains and also between species [108], even though phages exhibit the capacity to target these [109–111]. It is also known that phage resistance often comes at a cost to the bacteria, which can result in renewed sensitivity to antibiotics [112–116]. This trade-off could be further exploited through engineering. In terms of the treatment of intracellular pathogens, some of which are World Health Organisation Priority Pathogens [117], there has been some advancement towards improving the internalisation of phages, yet these may not be targeted to the vacuole where the bacteria are residing. Additionally, there is a lack of animal models in assessing phage efficacy. Phage therapy is unique in the fact that its clinical usage in humans was not preceded by *in vivo*animal models or clinical trials in humans, which is typically observed for most other drugs. This lack of background testing is due to phage therapy being largely employed under compassionate circumstances in the western world with tight time constraints not allowing animal testing. Therefore, it may be prudent to establish some level of *in vivo*efficacy via animal models before transitioning to clinical trials in humans. This may also provide opportunities to screen for adverse effects prior to clinical trials, especially for genetically modified phages. The development of phage therapeutics for widespread use and prescription will rely heavily on investment in clinical trials, as has been the case for antibiotics currently in clinical use. Such a task will require extensive knowledge of phage selection, applicability to the patient and pharmacodynamics going forward. This will also require a robust regulatory framework to support licensed and unlicensed use of phages for therapy. There have been propositions for the establishment of national phage banks, where scalable phage preparations will be produced on-site in accordance with national manufacturing practices with rigorous testing for safety and efficacy in conjunction with national healthcare services [31,118,119]. Readers are guided to the reviews by Jones et al. [31], Willy et al. [118] and Zalewska-Piątek [119], for current and comprehensive views on progressing phages from *ad hoc* to widespread usage in the Western world. As with any genetically modified organism, there has been some talk regarding the ramifications of these genetically modified phages being released into the wild and continuing to circulate or dominate the population [120]. However, the narrow phage host range means that it is unlikely that any phage released from the human host after treatment would be able to continue to propagate, making the spread of genetically modified phages far less of a risk than the spread of other genetically modified organisms. Time constraints for personalised therapies may also be hampering the translation of engineered phages into the clinic as engineering phages through any means is a time-consuming and difficult process. With most applications of phage therapy, being done in compassionate circumstances, time to treatment is of utmost importance. However, with the widespread creation of phage banks [30,121,122], there is room for the time to be taken to increase the engineered phages. It should be noted that despite all the successes of phage engineering within this review, countless more attempts have been made, met with failure and remain undocumented. Phage engineering is a difficult and time-consuming process requiring diverse strategies and extensive troubleshooting, even in archetypal phages such as T4 and T7. There are contradictions in the efficiency of engineering that reflect the complexity of the engineering processes, most often due to the low efficiency of the techniques employed. Despite improvements in this area with the advent of CRISPR–Cas9 engineering methodologies, extensive fine-tuning and optimisation are required to increase engineering efficiencies [123,124]. Intellectual property rights may also hinder engineering developments as these often take time to establish and this may result in a delay in these techniques being shared. Ultimately, there is the space for engineered phages with expanded host ranges [36] or for weaponised heads [41] to be produced and then used in the clinic and only time will tell if these enhanced phages rise to the challenge and become part of the standard treatment regime.

## Perspectives

- Phage therapy has become a strong weapon in the fight against antibiotic resistance. The engineering of phages enhances their capabilities to increase the benefit of the viruses.
- A variety of mechanisms have been deployed to improve phages, including increasing the host range, evolving them to increase their adaption for a particular host or environment and adding functionality through modified payload. Overall, these modifications appear to be stable and have positive outcomes for the clearance for bacterial infections.
- Despite advancements being made to individual phages, very few have been used in the clinic and only two clinical trials have included modified phages. Engineered phages represent great potential and work needs to be done to ensure increased usage of these phages in individual therapies, in clinical trials and in phage biobanks.

## Abbreviations

CRAB: Carbapenem-Resistant *Acinetobacter baumannii*
QS: quorum sensing
